# Correspondence

**Published:** 1883-04

**Authors:** Nathan Bozeman

**Affiliations:** 296 Fifth Avenue, New York


### CORRESPONDENCE.

### DR. BOZEMAN'S REJOINDER TO DR. MUNDE'S REPLY.

TO THE EDITOR OF THE ATLANTA MEDICAL REGISTER:

DEAR SIR—After carefully reading the reply of Dr. Munde, in the March issue of the REGISTER, to my criticism upon his statistics of "Prolapse of the Ovaries," which was made in the course of my discussion of "The Value of Graduated Pressure, etc.," I have come to the conclusion that the communication, from beginning to end, is so personal in spirit, so erratic in statement, so destitute of facts, and so entirely lacking in candor and fairness, as scarcely to require serious consideration.

Perhaps by way of caution to the readers of the REGISTER, to whom this young man is little known, less, perchance, some one should be deceived by his extraordinary claim of skill as a diagnostician, it is well for me to state that his merit rests largely upon his own estimation. He tells them in his reply that he differentiated normally situated from prolapsed ovaries in the proportion of 68 to

77 out of 145 palpated cases, which is *unprecedented* and *contrary* to the experience of the best writers of the day.

In my criticism of his statistics upon "Prolapse of the Ovaries," of which he now complains, I think I did him a service by unintentionally misquoting his figures, since from my statement it would be understood by any one familiar with the subject that he might possibly have encountered 145 cases of prolapsed and palpable ovaries out of a run of 1,600 unselected cases, provided the time of observation had been longer than nine months. Whereas, had his differentiation by palpation of 68 out of the 145 cases been stated by me as normally situated ovaries, the estimation and appreciation of his figures would have been quite another thing, even as the result of a lifetime observation and experience.

When Dr. Munde presumes to tell the readers of the REGISTER that he was familiar with the subject of prolapsed ovaries previous to September, 1878, when I submitted my paper to the American Gynæcological Society, giving prominence to the relationship of the affection to retro-displacements of the uterus, together with its scientific treatment, or that he was ignorant of my views upon the subject at the time of reading his paper on "Prolapse of the Ovaries" before the same Society, September, 1879, or that the principle of firmly columning the vagina with dry cotton in the form of pads possessed no distinctive difference from that of stuffing with dry wool "the entire vaginal canal from its vault to the floor of the pelvis, completely and compactly," as he had first learned it from another person, he simply shows his inability to prove the sources and genuineness of his statistics on the subject, and that he fails to recognize a true surgical principle when he sees it.

A man may zealously and blindly follow in the steps of another who knows no more of philosophy than himself. This will not make him a philosopher, nor will it prove that the man in whom he places his faith is not a pretender. Verily it is a small circle around which human knowledge usually travels, as well in medicine as in other branches of science.

In conclusion, I would add that Dr. Munde, after writing his reply for the REGISTER, opened a correspondence with me, for what reason it is best known to himself. Suffice it to say, that in this correspondence, extending through several days and comprising eight letters, all the facts relating to my criticism upon his statistics of "Prolapse of the Ovaries," as well as my reasons for making it, are fully brought out, as viewed from our respective standpoints. My reasons I do not propose to state here, further than to say they constitute some of the Doctor's "details which would have rendered the actual facts much more clear" in his reply, and which will, I trust, be a lesson to him in the future. Very respectfully,

NATHAN BOZEMAN.

296 Fifth Avenue, New York, March 15, 1883.

